# Oxidation in Low Moisture Foods as a Function of Surface Lipids and Fat Content

**DOI:** 10.3390/foods10040860

**Published:** 2021-04-15

**Authors:** Cansu Ekin Gumus, Eric Andrew Decker

**Affiliations:** 1 Department of Food Engineering, Ankara University, Ankara 06830, Turkey; cegumus@ankara.edu.tr; 2 Department of Food Science, University of Massachusetts Amherst, Amherst, MA 01003, USA

**Keywords:** lipid oxidation, low moisture food, surface lipid

## Abstract

Lipid oxidation is a major limitation to the shelf-life of low moisture foods and can lead to food waste. Little is known of whether the surface lipids in low moisture foods are more susceptible to oxidation since they are exposed to the environment. Therefore, the purpose of this research is to compare the rate of oxidation in surface and total lipids. Lipids in crackers were found to be in a heterogeneous matrix with proteins and starch, as determined by confocal microscopy. However, unlike spray-dried powders, both surface and interior lipids oxidized at similar rates, suggesting that the cracker matrix was not able to protect lipids from oxidation. Increasing the fat content of the crackers increased oxidation rates, which could be due to differences in the lipid structure or higher water activities in the high-fat crackers.

## 1. Introduction

Lipid oxidation has been studied extensively in various food matrices, such as bulk oils, emulsions, and meats [1,2,3,4,5]. However, the fundamental mechanisms of lipid oxidation in low-moisture foods (e.g., crackers, breakfast cereals, cakes, cookies, and baked goods) are much less understood, which is somewhat surprising since the shelf-life of many of these products is limited by oxidative rancidity [6]. Gaining a better understanding of the fundamental mechanism of lipid oxidation in these low moisture foods could help in taking a systematic approach to developing antioxidant strategies for this food category. If antioxidant technologies can be developed to prevent rancidity, then any oil can be utilized in these products.

Lipid oxidation, which decreases product quality and safety by forming off-aromas and degrading nutrients and potentially forming toxic compounds, starts taking place as soon as biological tissues are processed into foods; this oxidation continues during handling and storage [7]. Crackers and cookies are expected to have long shelf lives, but they will eventually spoil due to oxidative degradation [8]. The mechanisms of the rancidity do not follow the same route for bulk oils and fats as they do for baked goods due to the composition and physical structure of the baked goods [8].

Chemically leavened low moisture foods typically contain flour, fat, water, salt, and baking soda and/or baking powder. Solid fat is generally needed to help in the incorporation of air and to limit the formation of gluten to make the products tender and/or flaky. The leavening agent is another factor affecting cracker quality [9]. Commonly used leavening agents include sodium bicarbonate, ammonium bicarbonate, and monocalcium phosphate [9], and these additives tend to make the crackers alkaline.

Recent studies have helped researchers gain a better understanding of lipid oxidation in chemically leavened model crackers. Different from most other food systems, the lag phases of lipid hydroperoxide formation are much shorter than the lag phase of hydroperoxide breakdown products such as hexanal [10]. This suggests that pro-oxidants that decompose hydroperoxides are less active in chemically leavened crackers. A major pro-oxidant that decomposes hydroperoxides is transition metals; it was found that the addition of iron did not increase oxidation rates, and the presence of metal chelators did not decrease oxidation, suggesting that iron is not a major pro-oxidant in the chemically leavened crackers [10].

Another factor that impacts lipid oxidation in low moisture foods such as crackers is water activity. Quast and Karel (1972) studied the oxidation behavior of potato chips, representing moisture- and oxygen-sensitive foods, and showed that when a product was dried too much and water activity became too low, lipid oxidation reactions would increase [11]. In a chemically leavened system, Vu et al. (2020) found that lipid oxidation decreased with increasing water activity from 0.05 to 0.7 [12]. This was different from potato chips, which had the lowest oxidation rate at intermediate relative humidity (40% RH = water activity, aw = 0.4), with oxidation increasing with increasing relative humidity [11]. It is not clear why lipid oxidation reactions in cracker systems and potato chips respond differently to water activity, but one potential reason could be the physical location of the lipids. In the crackers, the lipids are incorporated into the dough, where they form a heterogeneous matrix with starch and protein. Conversely, in potato chips, the lipids adhere to the exterior of the product during cooking and also penetrate the potato cells upon removal of water during thermal processing.

A method used to study lipid oxidation as a factor of lipid location is to isolate surface or rapidly extractible lipids. Rapidly extractible lipids have been used to show that surface lipids are more susceptible to lipid oxidation in spray-dried powders compared to the lipids encased by wall materials in the powder interior during microencapsulation [13,14,15,16,17]. Drusch and Berg (2008) found that the extractible oil on the surface of dry powders will oxidize before internal fat and that the surface fat can be used to predict the shelf life of a product [17].

Another factor that has not been studied in low moisture foods is how the level of fat impacts lipid oxidation. In meat products, fat concentration has little impact on oxidation rates. This is because lipid oxidation in meat initially occurs in the cell membranes, and increasing fat concentration by the addition of adipose lipids (triacylglycerols) does not increase membrane lipid concentrations and, thus, has little impact on oxidation rates [18,19,20]. In oil-in-water emulsions, fat content can increase lipid oxidation rates if the fat concentration is increased while the particle size remains constant, as this will increase the surface area of the lipids [21]. Since oxidation of lipids in oil-in-water emulsions initially occurs at the emulsion droplet interface [22,23,24,25,26], this can increase oxidation rates.

The aim of this study is to determine the rate of lipid oxidation of surface versus total lipids to evaluate how lipid location impacts the oxidative stability of chemically leavened crackers. In addition, the impact of fat content on oxidation rates of low-moisture foods is investigated.

## 2. Materials and Methods

### 2.1. Materials

Interesterified soybean oil was provided by Archer Daniels Midland Company (Decatur, IL, USA) and kept at −20 °C until use. The interesterified soybean oil was composed of 11.1 weight (wt.) % palmitic acid, 22.2% stearic acid, 14.8% oleic acid, 43.3% linoleic acid, and 6.5% linolenic acid [12]. Baking soda (Arm & Hammer, York, PA, USA), iodized table salt (Morton, Chicago, IL, USA), and all-purpose flour (Pillsbury, Minneapolis, MN, USA) were purchased from local grocery stores. The nonpolar fluorescent dye BODIPY 493/503 was purchased from Invitrogen (Carlsbad, CA, USA). The fluorescent probe Rhodamine B and all other chemicals were purchased from Sigma-Aldrich (St. Louis, MO, USA). Double distilled water was used for all analyses.

### 2.2. Methods

**Figure 1:**
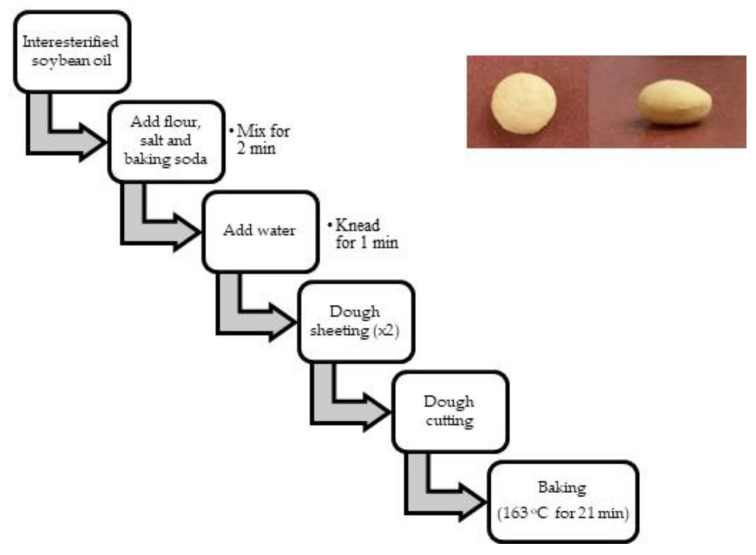
Flow diagram of cracker baking. Insert: whole crackers.

Cracker preparation (Figure 1) was adapted from Barden et al. (2015) [10]. A mixture of flour (50.6 wt. %), salt (1.2 wt. %), and baking soda (0.5 wt. %) was added to interesterified oil (8.1 wt. %), as shown in Table 1. Ingredients were mixed using a Kitchen Aid mixer (model #KSM95, Mississauga, ON, Canada) for 2 min. Water (31.6 wt. %) was added, and the resulting dough was kneaded by hand with the incorporation of additional flour (8.1 wt. %) to minimize sticking. The dough was flattened by a pasta roller (Kitchen Aid KPSA attachment, Mississauga, ON, Canada; thickness setting 2, approximately 3 mm). For the surface oil analysis, the opening of a disposable test tube (13 × 75 mm, Fisher Scientific, Hampton, NH, USA) was used to cut the dough into small round pieces that could be used to extract surface lipids. To evaluate the effect of lipid concentration on lipid oxidation and microstructure, the dough was cut into 2.5 × 2.5 cm pieces before baking. All crackers were baked at 163 °C for 21 min. In the latter experiments, the fat content of the crackers was changed by altering the fat and water content while keeping all other ingredient concentrations the same. Table 1 shows the fat and water concentrations.

For confocal imaging, a Rhodamine B stock solution was prepared by dissolving 1 g Rhodamine B in 15 mL ethanol. This hydrophilic fluorescent dye solution (40 µL) was added to the water prior to mixing it with other cracker ingredients. BODIPY 493/503 stock solution was prepared by dissolving 10 mg dye in 15 mL ethanol:chloroform solution (2:1). Lipophilic BODIPY (2.7 µg BODIPY 493/503/g lipid) was added to the fat prior to mixing.

After baking, all crackers were allowed to cool to room temperature before handling. For the surface oil analysis, whole (round) crackers were directly placed in 10 mL glass gas chromatography (GC) vials (Supelco Analytical; Bellefonte, PA, USA). For the evaluation of the effect of lipid concentration on lipid oxidation, the 2.5 × 2.5 cm square crackers were crumbled using a mortar and pestle, distributed (0.5 g) in 10 mL volume glass headspace vials, and sealed with silicone/polytetrafluoroethylene (PTFE) septa screw caps for the storage studies. Vials were incubated at 55 °C in corrugated cardboard containers. The use of light-protected carriers was crucial to avoid the increased effects of ultraviolet–visible (UV/vis) radiation on lipid oxidation rates. Incubation was a maximum of 45 days, with testing occurring every few days.

*Confocal Microscopy Analysis*: A Nikon confocal microscope (C1 Digital Eclipse, Tokyo, Japan) with a PL FLUOTAR ELWD 20.0 × 0.45 objective lens was used to capture the confocal images in intact 2.5 × 2.5 cm crackers. Crackers containing both BODIPY and Rhodamine B were imaged sequentially, and the resulting images were overlaid. An air-cooled argon-ion laser (Model 376 IMA1010 BOS; Melles Griot; Carlsbad, CA, USA) was used to excite BODIPY at 488 nm, and emission spectra were collected at 515 ± 30 nm. A Melles Griot helium–neon laser (Model 05-381 LGP-193; Carlsbad, CA, USA) was used to excite Rhodamine B at 543 nm, and emission spectra were collected at 605 ± 75 nm. Detector pinhole size was always 150 μm. All resulting images consisted of 512 × 512 pixels, with a pixel size of 414 nm and a pixel dwell time of 10.40 μs. Confocal microscopy images were analyzed using EZ-CS1 (version 3.8) software (Nikon; Melville, NY, USA).

*Water activity measurement*: Crushed samples were used to measure water activity using a Decagon Devices AquaLab Series 3 water activity meter (Pullman, WA, USA).

*Lipid isolation*: For the evaluation of the effect of lipid concentration on lipid oxidation, the crushed crackers (0.105 ± 0.001 g) were extracted with 5 mL chloroform:methanol solvent (2:1 *v/v*) by vortexing for 30 s, followed by centrifuging (Centrific TMCentrifuge, Thermo Fisher Scientific Inc., Fairlawn, NJ, USA) at 3400× *g* for 10 min and isolation of the solvent phase. On the other hand, for surface oil analysis, two whole crackers that weighed 0.5 ± 0.05 g each were selected and extracted with chloroform:methanol solvent (2:1 *v/v*) at the ratio of 1:7.5 (cracker:solvent) by gently shaking for 1 min. All lipid concentrations were normalized using actual sample weights.

*Primary lipid oxidation products*: The Shantha and Decker (1994) method was used to monitor lipid hydroperoxide formation [27]. This method uses a modified version of the International Dairy Federation method. After lipid extraction, the solvent phase (200 µL) was mixed with 16.7 µL of 1:1 FeSO_4_ (in water):BaCl_2_ (in 0.4 N HCl) solution. Then, 16.7 µL of NH_4_SCN in water was added. The absorbance was measured at 500 nm after 20 min using a Genesys 20 spectrophotometer (ThermoSpectronic; Waltham, MA, USA). A standard curve was prepared with cumene hydroperoxide.

*Secondary lipid oxidation products*: The formation of a secondary oxidation product, hexanal, was monitored by a GC-2014 Shimadzu gas chromatograph equipped with an AOC-5000 autosampler (Shimadzu; Kyoto, Japan) using the method by Pignoli et al. (2009) [28]. A 50/30 μm divinylbenzene/carboxen/polydimethylsiloxane (DVB/Carboxen/PDMS) solid-phase microextraction (SPME) fiber (Supelco, Bellefonte, PA, USA) was inserted through the vial septum and exposed to the sample headspace for 10 min at 55 °C. The SPME fiber was desorbed at 250 °C for 3 min in the gas chromatograph detector at a split ratio of 1:7. The temperatures of the oven, injector, and flame ionization detector were 65, 250, and 250 °C, respectively, and the samples ran for 6 min. Volatile aldehydes were separated on a fused-silica capillary column (30 m × 0.32 mm internal diameter × 1 μm) coated with 100% PDMS (Equity-1, Supelco, Bellefonte, PA, USA), and peak integration was calculated using Shimadzu EZstart (version 7.4) software. A standard curve was prepared by using a standard hexanal solution in methanol added to crackers, and concentrations of hexanal in the samples were calculated using this curve.

*Statistical analysis*: All experiments were conducted in triplicate. All calculations compared the means by analysis of variance (ANOVA) and used a significance level of *p* < 0.05. Lag phases for the oxidation studies were identified as Day 1, which had significantly higher (>0.05) concentrations of oxidation products than Day 0. All calculations were performed using IBM SPSS Statistics 20 statistical software.

## 3. Results

### 3.1. Differences in Lipid Oxidation between Surface and Total Lipids

**Figure 2:**
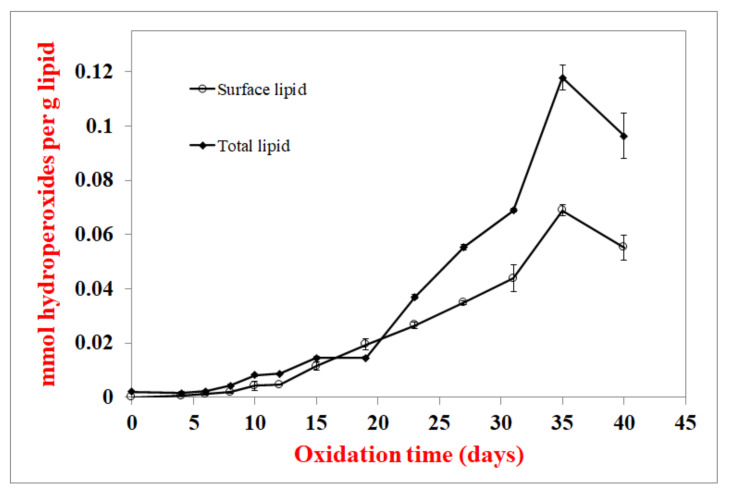
Surface oil vs. total lipid hydroperoxide formation at 55 °C. Data points are the average of 3 measurements ± standard deviations.

In the first experiment, whole crackers were incubated and, periodically, the surface and total lipids were extracted; lipid hydroperoxides were determined to see if the surface lipids oxidize before total lipids as they do in spray-dried powders. In order to extract the surface fat from dried powders, various techniques that use different solvents (hexane, butanol, chloroform, isopropanol, and methanol), different extraction times, and different sample-to-solvent ratios have been studied [13,14,15,16,17]. The ideal system would constantly extract just enough fat to measure oxidation products, assuming that the short extraction time would primarily extract surface lipids. Extracting with chloroform:methanol (2:1) mixture for 1 min was found to extract enough lipid (1.58 ± 0.05% of total cracker; 16 mg ± 0.6 mg lipid/g cracker; 12.51 ± 0.37% of total lipid in the cracker sample) to conduct hydroperoxide analyses. Headspace analyses were not conducted as volatile compounds would be lost during extraction. The lag phase for lipid hydroperoxide formation for both surface and total lipids was 8 days (Figure 2), indicating that oxidation rates were similar. Total lipids had higher hydroperoxide concentrations after 18 days, but this is not relevant to food quality and shelf life since both surface and interior lipids would be rancid by this time because sensory detection of oxidation products occurs shortly after the lag phase. Lipid hydroperoxide concentrations decreased in the later stages of storage, which is common as lipid hydroperoxide formation becomes slower than hydroperoxide breakdown upon extensive storage [29,30,31,32,33,34].

**Figure 3:**
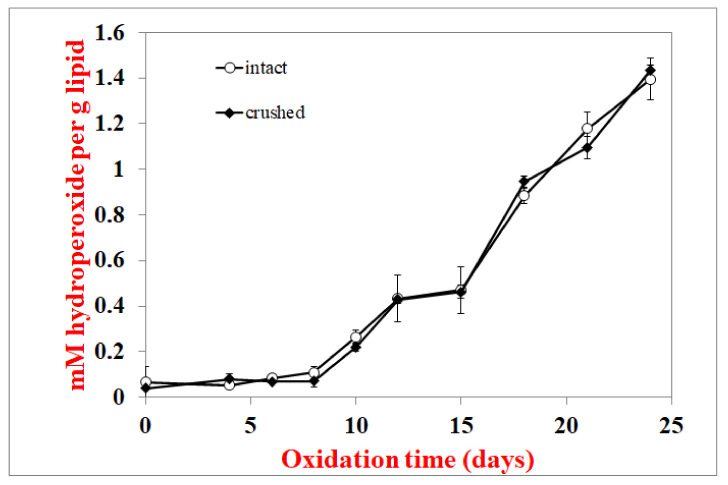
Lipid hydroperoxide formation at 55 °C in crushed and whole crackers. Data points are the average of 3 measurements ± standard deviations.

**Figure 4:**
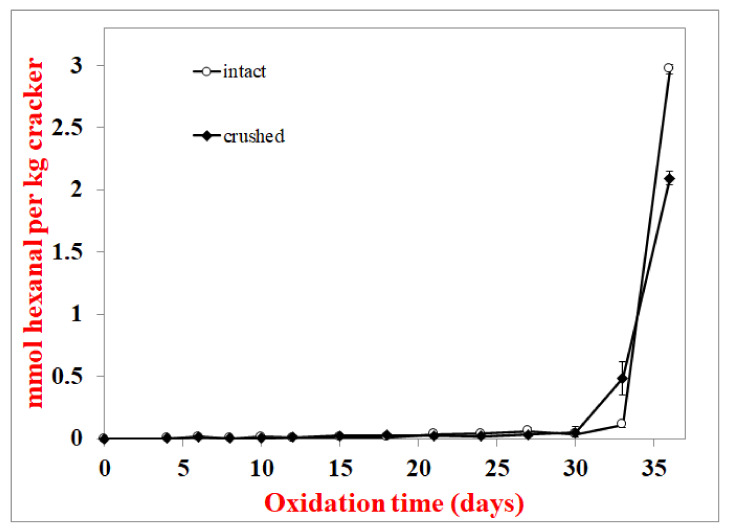
Hexanal formation in at 55 °C in crushed and round crackers. Data points are the average of 3 measurements ± standard deviations.

To verify that surface lipids do not oxidize faster than interior lipids, an experiment where crackers were stored intact (whole) or after they were crushed with a mortar and pestle was also conducted. Crushing the crackers alters the physical structures that protect the interior lipids, and if this were the case, one would expect the crushed crackers to oxidize faster than the whole crackers. When crackers with 8.1% fat (similar to the fat content in commercial crackers) were stored as whole or crushed, the lag phase of lipid hydroperoxide formation was the same at 8 days (Figure 3). Since these crackers were not subjected to solvent extraction, headspace hexanal could be monitored as well. The hexanal lag phase was 24 and 27 days for the whole and crushed crackers, respectively (Figure 4). Since hexanal formation was faster in whole than crushed crackers, this suggests that disrupting the cracker structure did not expose the interior lipids in a way that they would be more susceptible to oxidation.

### 3.2. Effect of Fat Concentration on Lipid Oxidation

**Figure 5:**
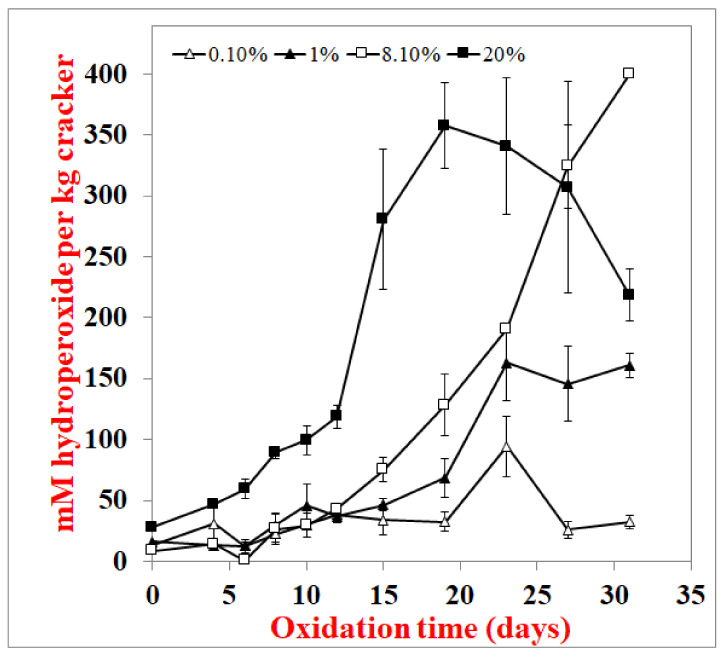
Lipid hydroperoxide formation in crackers with different fat contents at 55 °C. Data points are the average of 3 measurements ± standard deviations.

**Figure 6:**
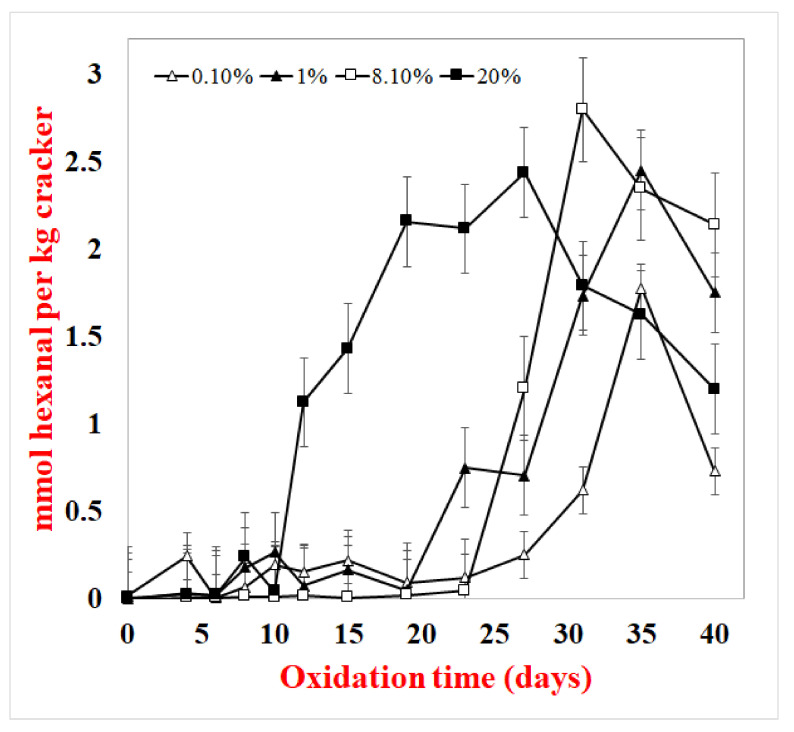
Hexanal formation in of crackers with different fat contents at 55 °C. Data points are the average of 3 measurements ± standard deviations.

Crackers made with varying fat concentrations (0.1–20%) were also examined as increasing fat content could potentially alter the location of the lipids and/or make it more difficult for the cracker matrix to encapsulate and protect the lipids. In the 20% fat crackers, the hydroperoxide lag phase was 4 days, and the lag phase for hexanal formation was 10 days (Figure 5 and Figure 6). Hydroperoxide lag phases for the 1% and 8% fat crackers were longer at 10 days, and hexanal lag phases were 19 and 24 days, respectively. The hydroperoxide and hexanal lag phases for the 0.1% fat crackers were 19 and 24 days, respectively. The varied fat concentrations were produced by substituting fat for water in the cracker formulation. This resulted in decreasing water activity, with increased fat concentrations from 0.65 for 0.1% fat to 0.10 for 20% fat (Table 2).

**Figure 7:**
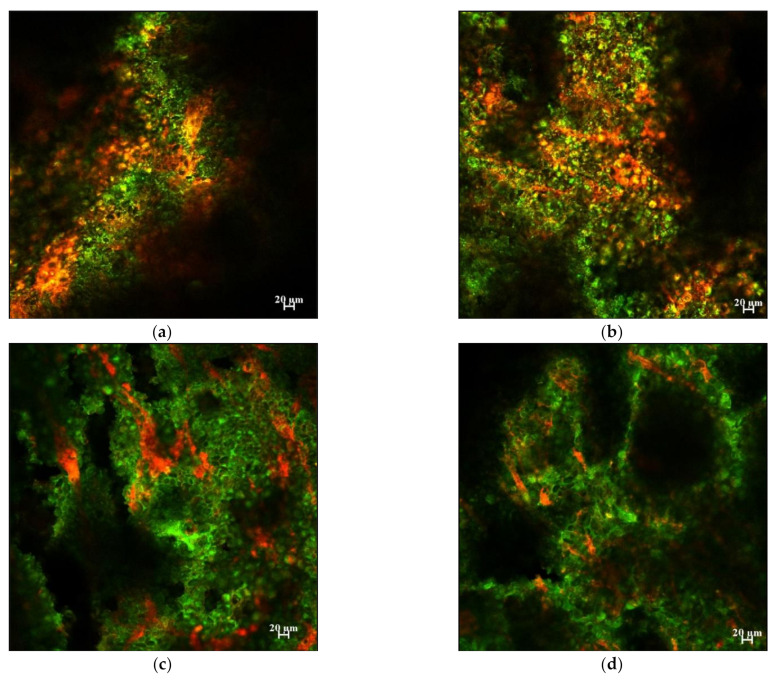
Confocal microscopy images of the crackers with (**a**) 0.1%, (**b**) 1.0%, (**c**) 8.1%, and (**d**) 20.0% fat content. Proteins are stained with Rhodamine B (seen in red), and lipid phases are stained with BODIPY (seen in green).

Confocal images were obtained from the crackers with different fat content (Figure 7). In these images, orange represents the proteins, green represents the lipids, and the black oval shapes represent the starch [10,35]. As fat concentration increased, the images of the protein and starch granules decreased, suggesting that the fat was coating the other flour components.

## 4. Discussion

### 4.1. Differences in Lipid Oxidation between Surface and Total Lipids

Hydroperoxide lag phases were the same for total and surface lipids in whole crackers (Figure 2). They were also similar for hydroperoxide and hexanal lag phases in whole vs. crushed crackers, where the crushing damages the cracker’s physical structure and should increase lipid exposure (Figure 3 and Figure 4). These two experiments suggest that the surface lipids in crackers are not oxidizing faster than interior lipids. Confocal microscopy can be a useful way to determine the physical state of lipids. Unlike spray-dried powders, where lipids are either on the surface or embedded in a glassy protein/carbohydrate matrix, the confocal images (Figure 7) showed that the lipids in the cracker were in a matrix of lipids and protein and not encapsulated. Since the lipids are not encapsulated, this can explain why the surface and interior lipids oxidize at the same rate. In addition, visual observation and the large black area in the confocal images showed that the crackers were very porous vs. the compact structures seen in spray-dried powders. This would mean that oxygen is present in both the interior and exterior of the crackers, allowing all lipid locations to oxidize at similar rates.

### 4.2. Effect of Fat Concentrations on Lipid Oxidation

When lipid oxidation was examined as a function of fat concentration, both hydroperoxide (Figure 5) and hexanal (Figure 6) formations increased with increasing fat content. Confocal imaging (Figure 7) showed that the fat increasingly masked the images of both the starch (black oval images) and protein (orange) as fat content increased [10]. This suggests that the high-fat concentrations were producing a thick continuous fat layer around the starch and protein, whereas the fat was more dispersed within the fat and starch at low-fat levels. The increased oxidation rates observed in the high-fat crackers could be due to the lipid acting more like a bulk lipid, where free radicals can more easily diffuse and oxidize unsaturated fatty acids. However, since crackers with increased lipids were made by replacing water with fat, this resulted in different water activity rates, with water activity decreasing from 0.65 to 0.10 from 0.1% to 20% fat. Vu et al. (2020) found that decreasing water activity increased oxidation rates in this cracker model [12]. This could also help explain the shorter hydroperoxide and hexanal lag phases in the high-fat crackers.

## 5. Conclusions

These experiments indicate that the surface lipids of crackers are not more susceptible to lipid oxidation than interior lipids. This suggests that all lipids are similarly exposed to oxygen and that antioxidant strategies focused on the protection of surface lipids are not likely to be successful. This could be due to the highly porous nature of the crackers, where oxygen is dispersed through the cracker. Higher fat content decreased oxidative stability, suggesting that lipid oxidation can be decreased by simply decreasing the fat level. Since the high-fat crackers have lower water activity, this also explains why they are less oxidatively stable, which suggests that any strategies to increase water activity can increase shelf life. Additional research is needed to understand how antioxidant technologies can be customized to better control lipid oxidation in low-moisture foods. This could include work with novel antioxidants or combinations of antioxidants that show synergistic activity. In addition, technologies such as modified atmospheres can be used to increase the efficacy of added antioxidants. If such technologies can be identified, this would significantly decrease food waste from the development of oxidative rancidity.

## Figures and Tables

**Table 1:** Ingredients used for cracker baking (g).

Ingredient	0.1% Fat	1.0% Fat	8.0% Fat	20% Fat
Flour	62.5 (50.6%)	62.5 (50.6%)	62.5 (50.6%)	62.5 (50.6%)
DI Water	48.82 (39.5%)	47.82 (38.7%)	39 (31.6%)	24.30 (19.7%)
Interesterified Soybean Oil	0.18 (0.1%)	1.18 (1.0%)	10 (8.1%)	24.70 (20.0%)
Salt	1.5 (1.2%)	1.5 (1.2%)	1.5 (1.2%)	1.5 (1.2%)
Baking Soda	0.58 (0.5%)	0.58 (0.5%)	0.58 (0.5%)	0.58 (0.5%)
Flour for sprinkling on surface	10 (8.1%)	10 (8.1%)	10 (8.1%)	10 (8.1%)
TOTAL	123.58 g	123.58 g	123.58 g	123.58 g

**Table 2:** Water activity of crackers with varying fat content at room temperature. Data points are the average of 3 measurements ± standard deviations.

Group	Water Activity ± Standard Deviation
0.1% fat	0.65 ± 0.04
1.0% fat	0.23 ± 0.02
8.0% fat	0.15 ± 0.03
20% fat	0.10 ± 0.01
